# Supplementary material for: Robust encoding of natural stimuli by neuronal response sequences in monkey visual cortex
Source: Nat Commun. 2023 May 25;14:3021. doi: 10.1038/s41467-023-38587-2 (PMC10212951; doi:10.1038/s41467-023-38587-2)
Supplement: Supplementary file 1 — Supplementary Information [file 41467_2023_38587_MOESM1_ESM.pdf]

Robust Encoding of Natural Stimuli by Neuronal Response Sequences in Monkey Visual Cortex

Supplementary Information

Yang Yiling, Katharine Shapcott, Alina Peter, Johanna Klon-Lipok, Huang Xuhui, Andreea Lazar, Wolf Singer

Supplementary Figures

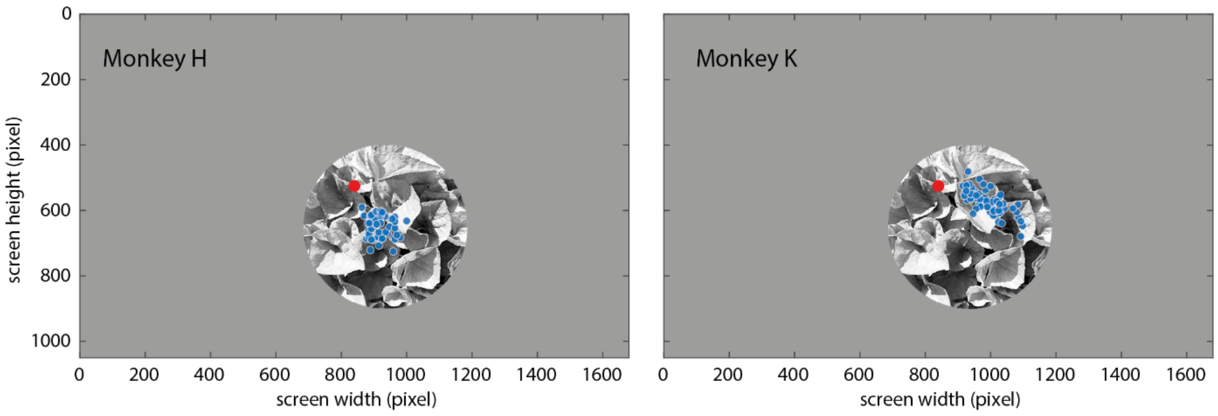

Supplementary Figure 1 Schematic of stimulus position and V4 classical receptive fields on the display monitor.

Left: monkey H. Right: monkey K. Red dot indicates the fixation point. Blue dots mark the receptive field centers. The stimulus picture covers both the fixation point and the receptive fields.

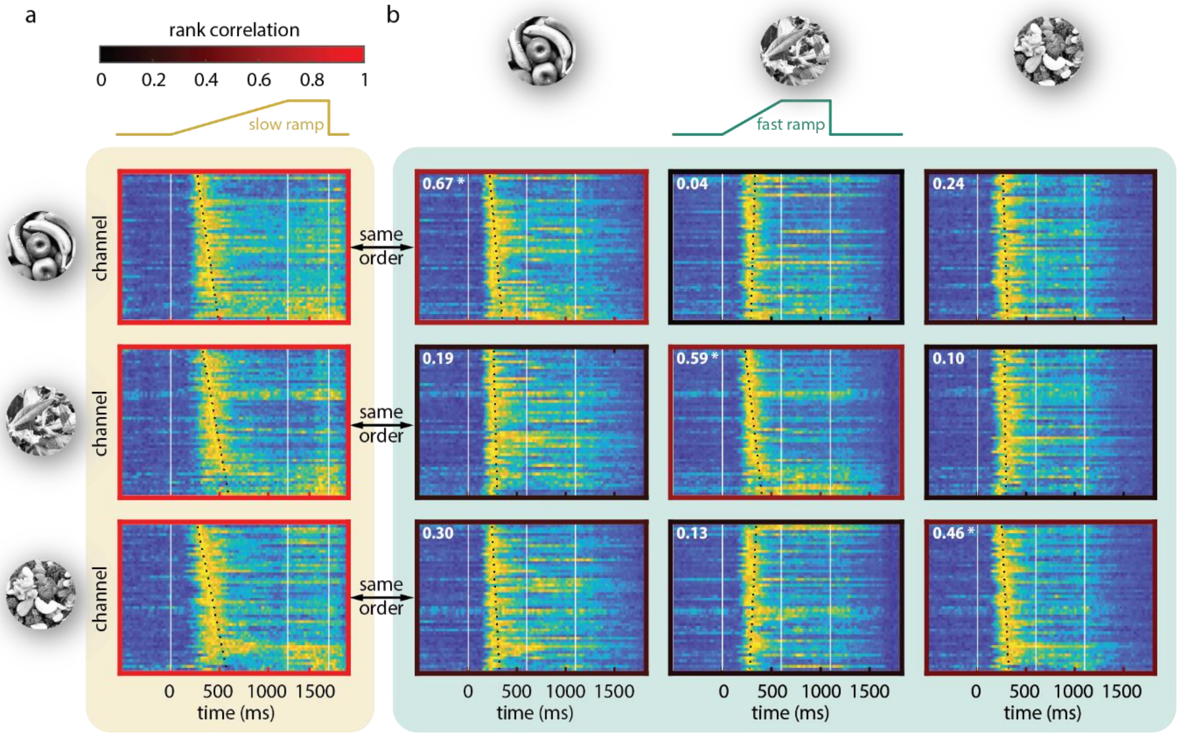

Supplementary Figure 2 Ramping stimulus intensity reveals scalable and stimulus-specific response sequences. Results from Monkey H. a & b: same conventions as in Figure 1 c & d.

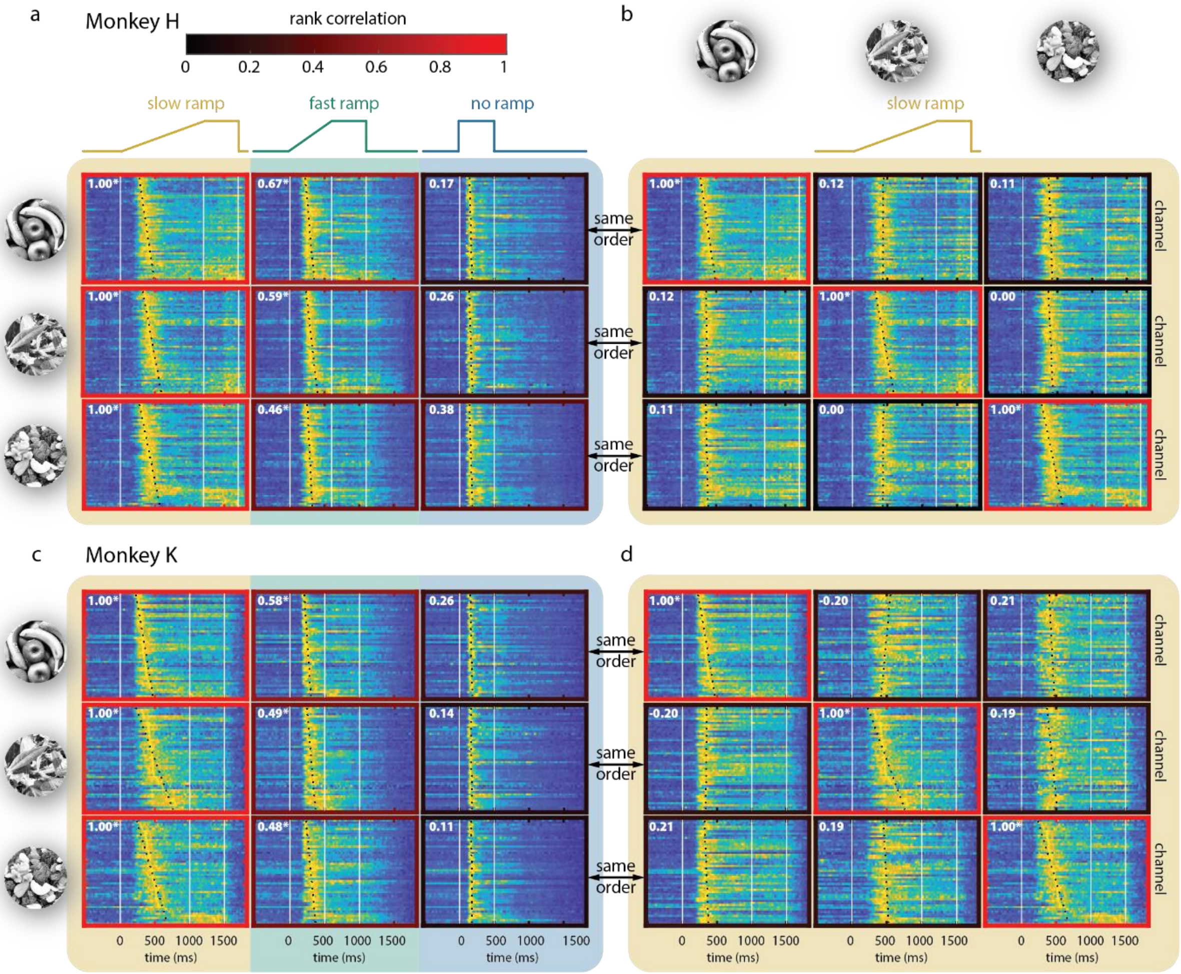

Supplementary Figure 3 Ramping stimulus intensity reveals scalable and stimulus-specific response sequences. Other examples from monkeys H and K.

a & b. Same convention as in Figure 1 c & d. For the panels in the same row in a and b, the firing rate responses are sorted in the same way, i.e. according to the rank order derived from the sequences evoked by the respective stimulus (indicated on the left in **a**) in the *slow* ramp condition. Results from monkey H. c & d. Same conventions as in a & b. Results from monkey K.

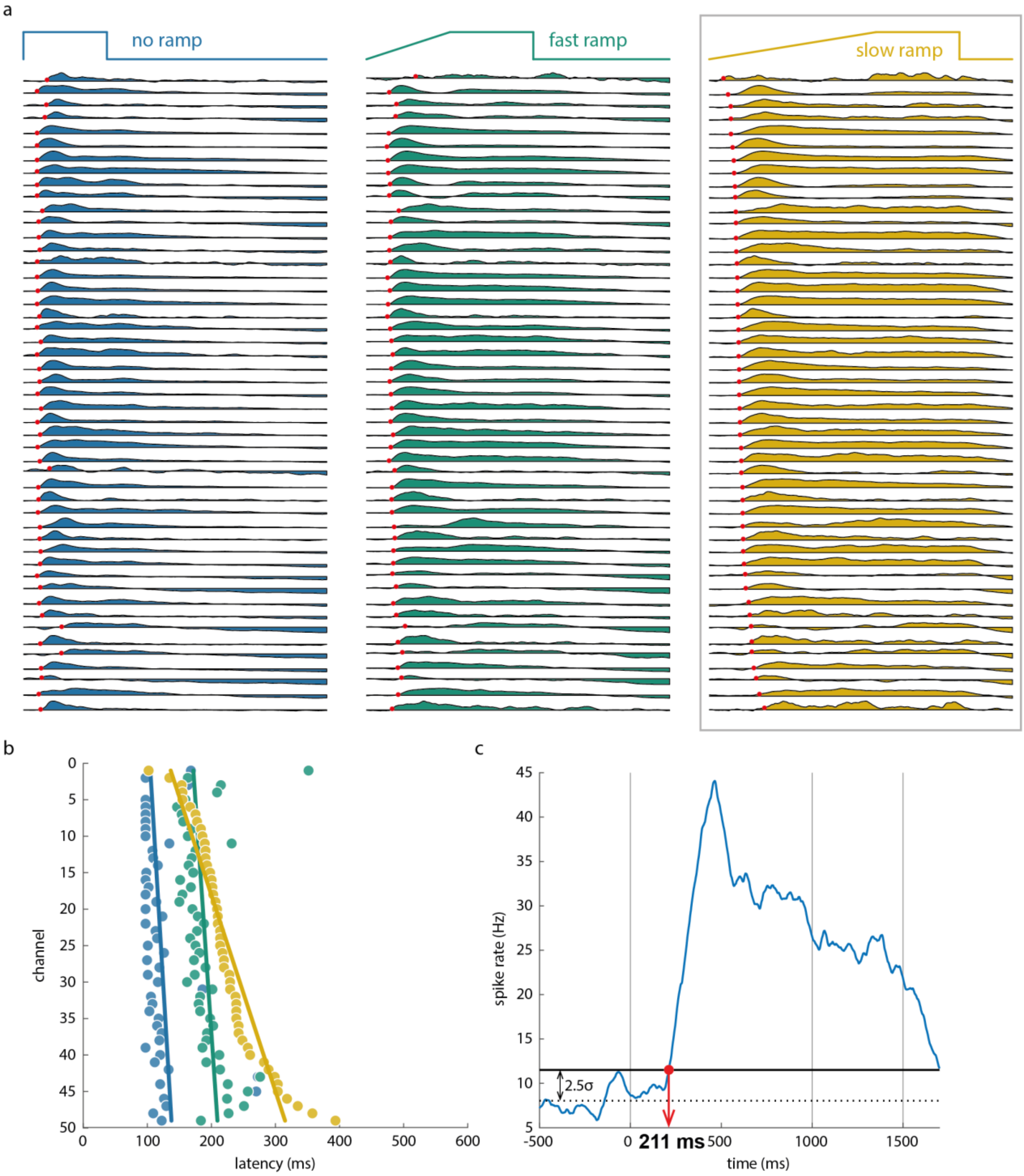

Supplementary Figure 4 V4 responses rank-ordered according to onset latencies.

a. Firing rate responses from different electrode channels (rows) evoked by a stimulus. The three columns refer to no ramp (left), fast ramp (middle) and slow ramp (right) conditions. All channels are sorted based on response onset latencies (marked by red dots) in the slow ramp condition (enclosed by gray box). b. Correlations between onset latencies and channel ordering. The correlation is significant for slow ramp ($r = 0.94$, $p < 0.01$) and fast ramp ($r = 0.30$, $p = 0.036$) conditions, but not for the no ramp condition ($r = 0.27$, $p = 0.065$). **c**. Schematic representation of the latency detection method. The threshold (black solid line) is set as baseline firing rate (dotted line) plus 2.5 times its standard deviation. Vertical grey lines denote stimulus onset, ramp end, and stimulus offset, respectively.

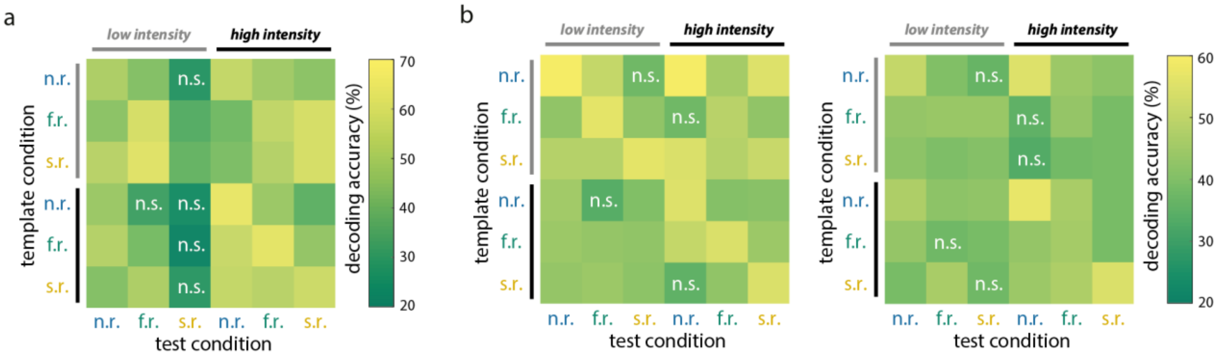

Supplementary Figure 5 Rank order of neuronal responses enable identification of stimulus-specific information.

a. Accuracy and generalization performance of decoding stimulus identity based on response rank orders. Same conventions as for Figure 1e, but for Monkey K (49.74 ± 1.96 %, t_{35} = 25.22, p = 4.94 × 10^{-24}). b. Similar to **a** but onset latencies rather than peak latencies were used to assess rank orders. Left and right panels are results for monkey H (47.11 ± 1.20 %, t_{35} = 39.11, p = 1.78 × 10^{-30}) and monkey K (42.97 ± 0.89 %, t_{35} = 47.79, p = 1.82 × 10^{-33}), respectively. Source data are provided as a Source Data file.

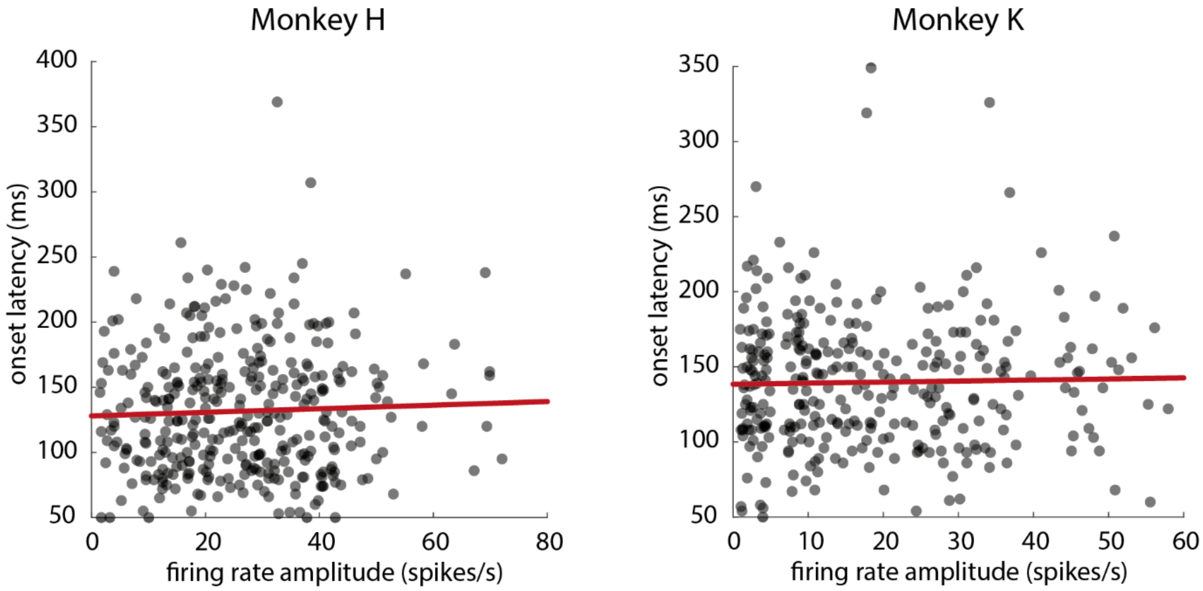

Supplementary Figure 6 Correlation between onset latency and firing rate amplitude.

Left: monkey H, r = 0.041, p = 0.18. Right: monkey K, r = 0.023, p = 0.48. Source data are provided as a Source Data file.

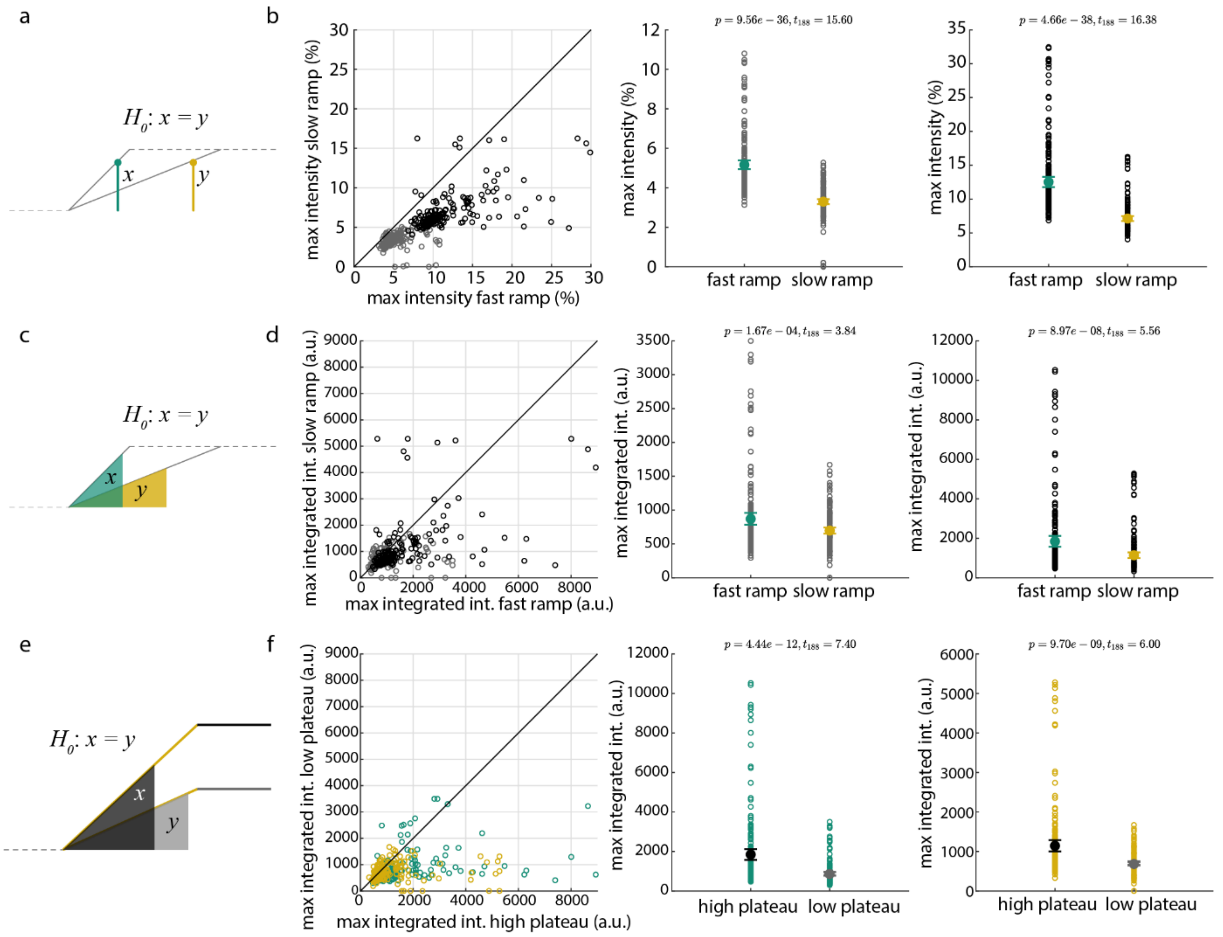

Supplementary Figure 7 Differences in neurons' sensitivity to stimulus energy can alone not account for the emergence of response sequences (results from monkey H).

a. Null hypothesis: the stimulus intensity at which a neuron responds maximally (maximal intensity) is equal in fast ramp (*x*) and slow ramp (*y*) conditions. b. Left: for each channel, maximal intensity in the fast ramp (*x*) condition is plotted against that in slow ramp condition (*y*, Left). Light and dark colors denote low and high intensity conditions, respectively. Middle and right panels: comparisons between ramp conditions (test of null hypothesis), separately for low and high intensity conditions, respectively. The *t* statistics (two-sided) and *p* values are inserted at the top of the figures. Error bars denote the 95% confidence interval around the mean (same below). c. Null hypothesis: the integrated input drive (in this case, the integral of stimulus intensity) at which a neuron fires maximally (maximal integrated intensity) is equal in fast ramp (*x*) and slow ramp (*y*) conditions. d. Left: Each channel's maximal integrated intensity in the fast ramp condition is plotted against that in the slow ramp condition. Middle and right panels: test of the null hypothesis (two-sided t-test). e. Null hypothesis: maximal integrated intensity is equal in high (*x*) and low (*y*) intensity conditions, given the same ramp duration. f. Left: For each MUA response, the corresponding integrated intensity is plotted for high against low intensity conditions. Middle and right panels: test of the null hypothesis (two-sided t-test). Green and yellow denote fast and slow ramp durations, respectively.

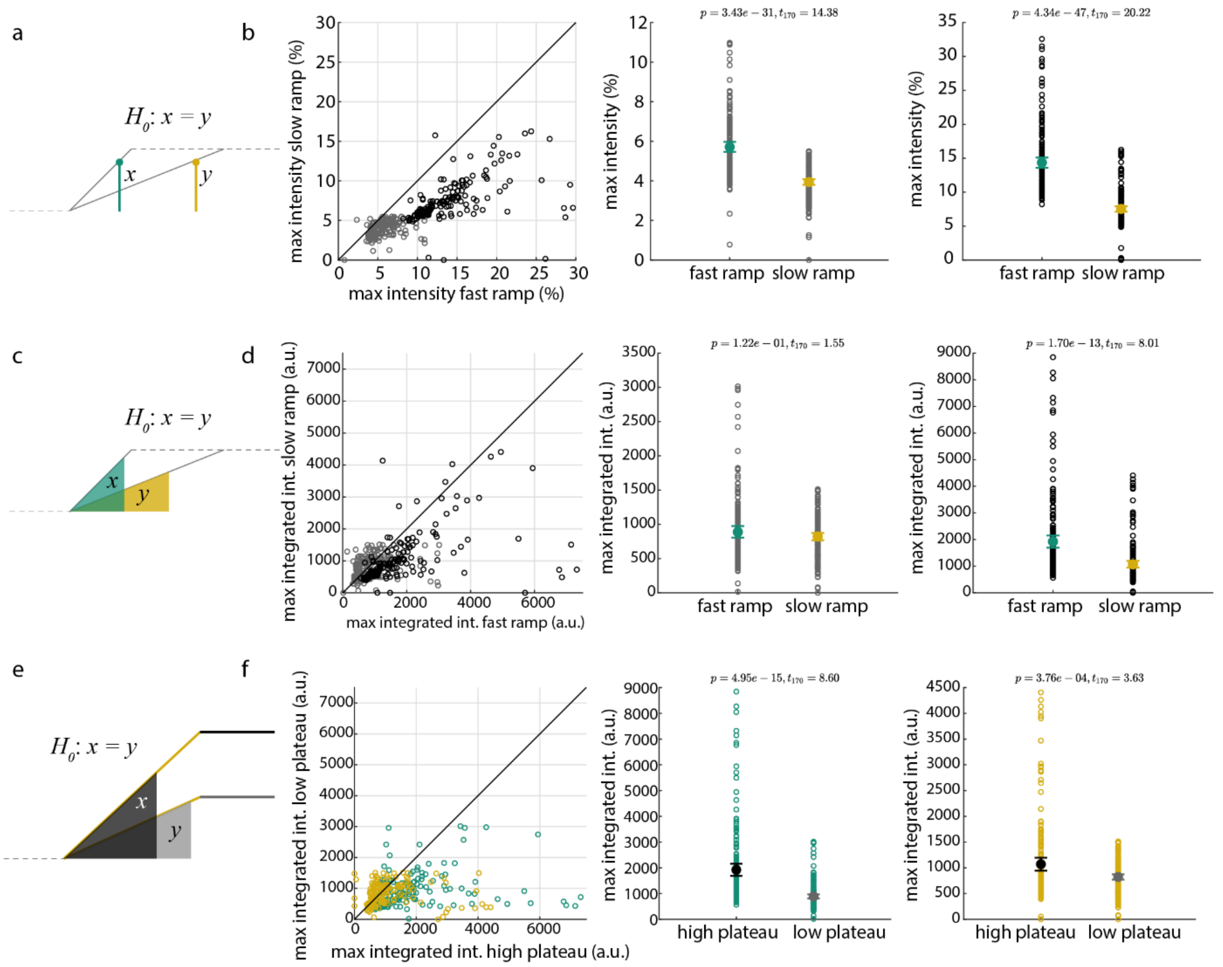

Supplementary Figure 8 Difference in multi-unit's sensitivity alone cannot explain the sequential response. a – f: same conventions as in **Error! Reference source not found.** a – f, but for monkey K.

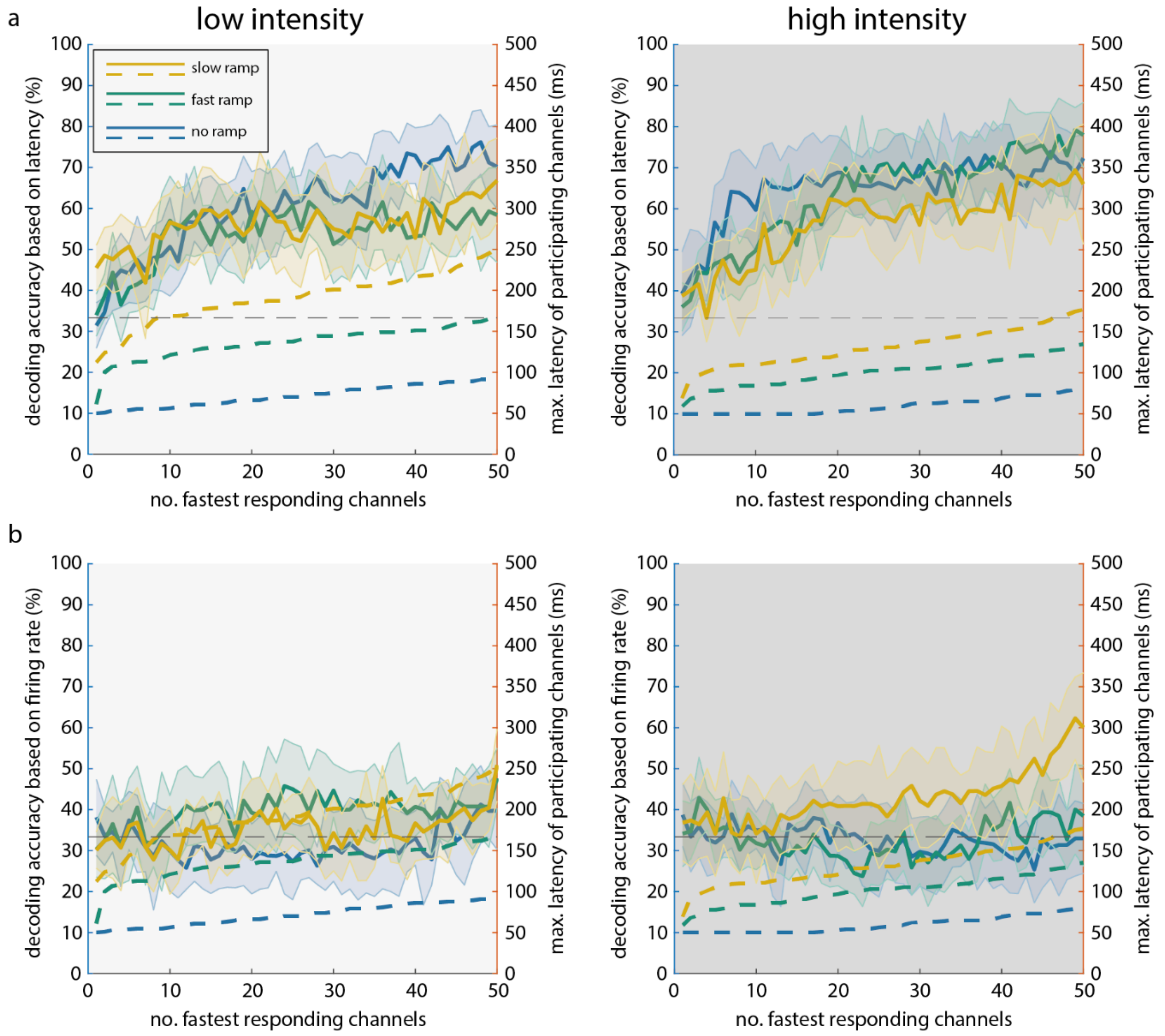

Supplementary Figure 9 Sequential neuronal responses enable fast identification of stimulus-specific information. a & b: same conventions as in Figure 2, but for monkey H. Source data are provided as a Source Data file.

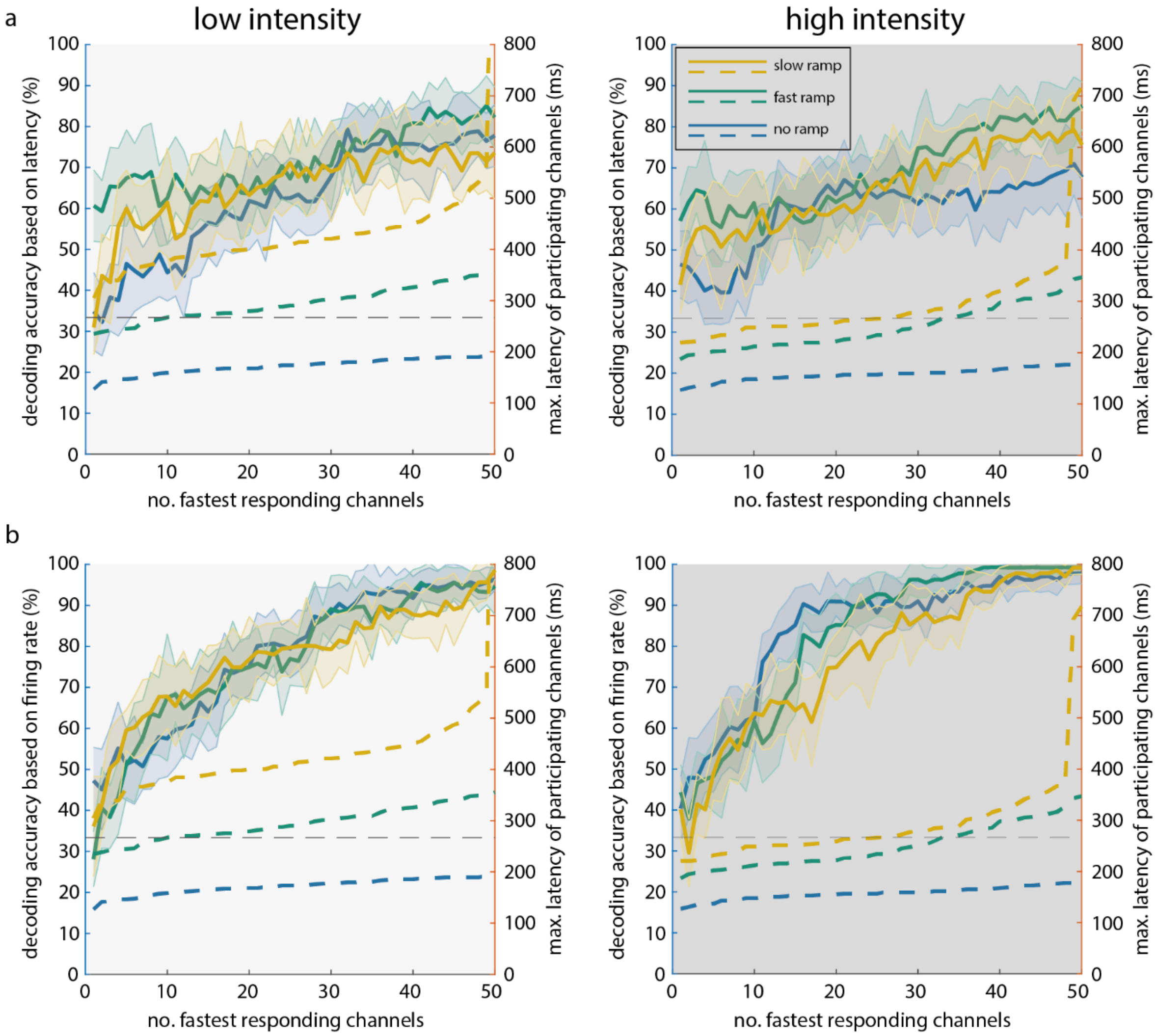

Supplementary Figure 10 Sequential neuronal responses enable fast identification of stimulus-specific information. a & b: same convention as in Figure 2, but response peak times rather than onset latencies were used in the analysis. Results from monkey H. Source data are provided as a Source Data file.

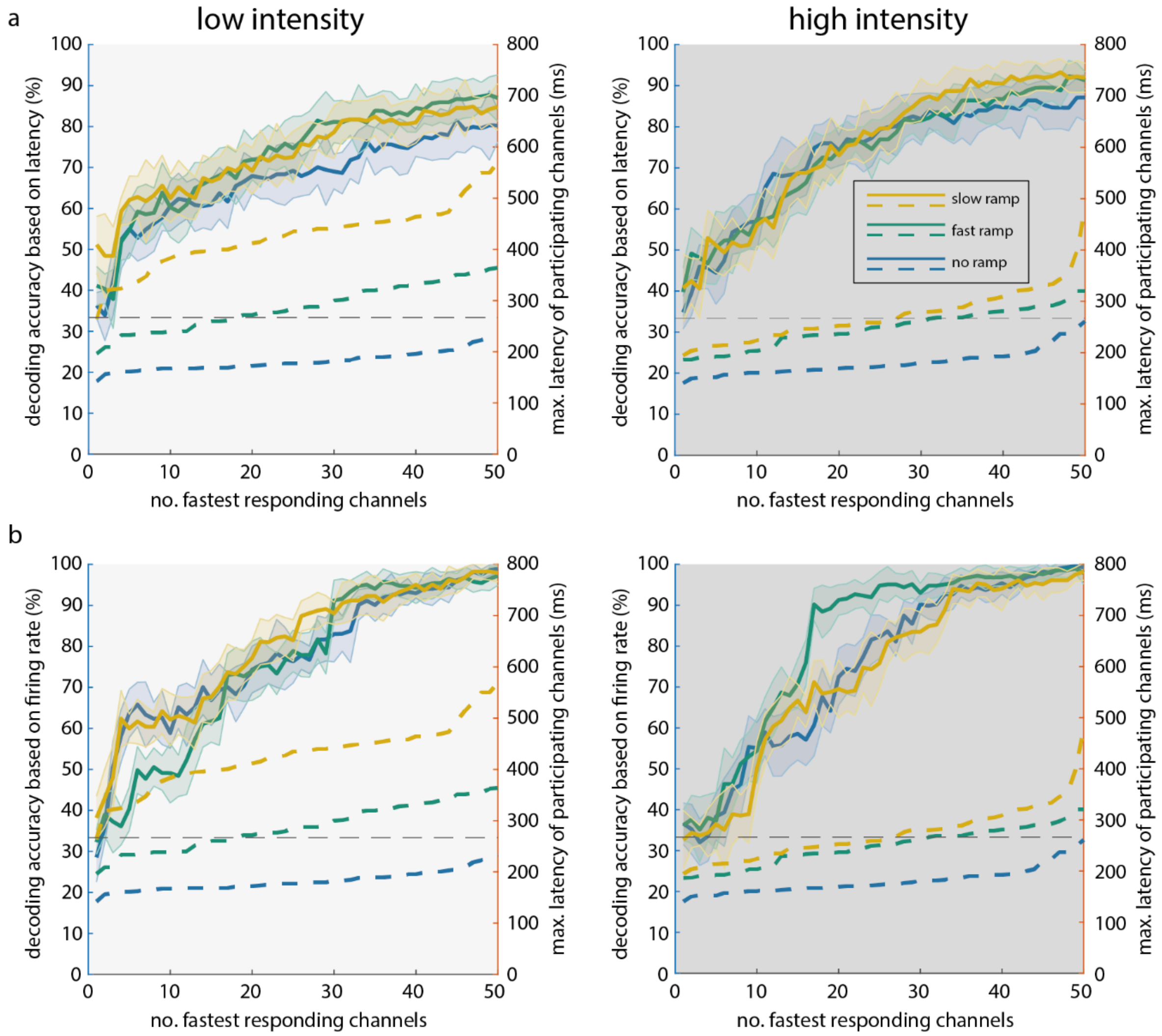

Supplementary Figure 11 Sequential neuronal responses enable fast identification of stimulus-specific information. a & b: same convention as in Figure 2, but response peak latencies rather than onset latencies were used in the analysis. Results from monkey K. Source data are provided as a Source Data file.

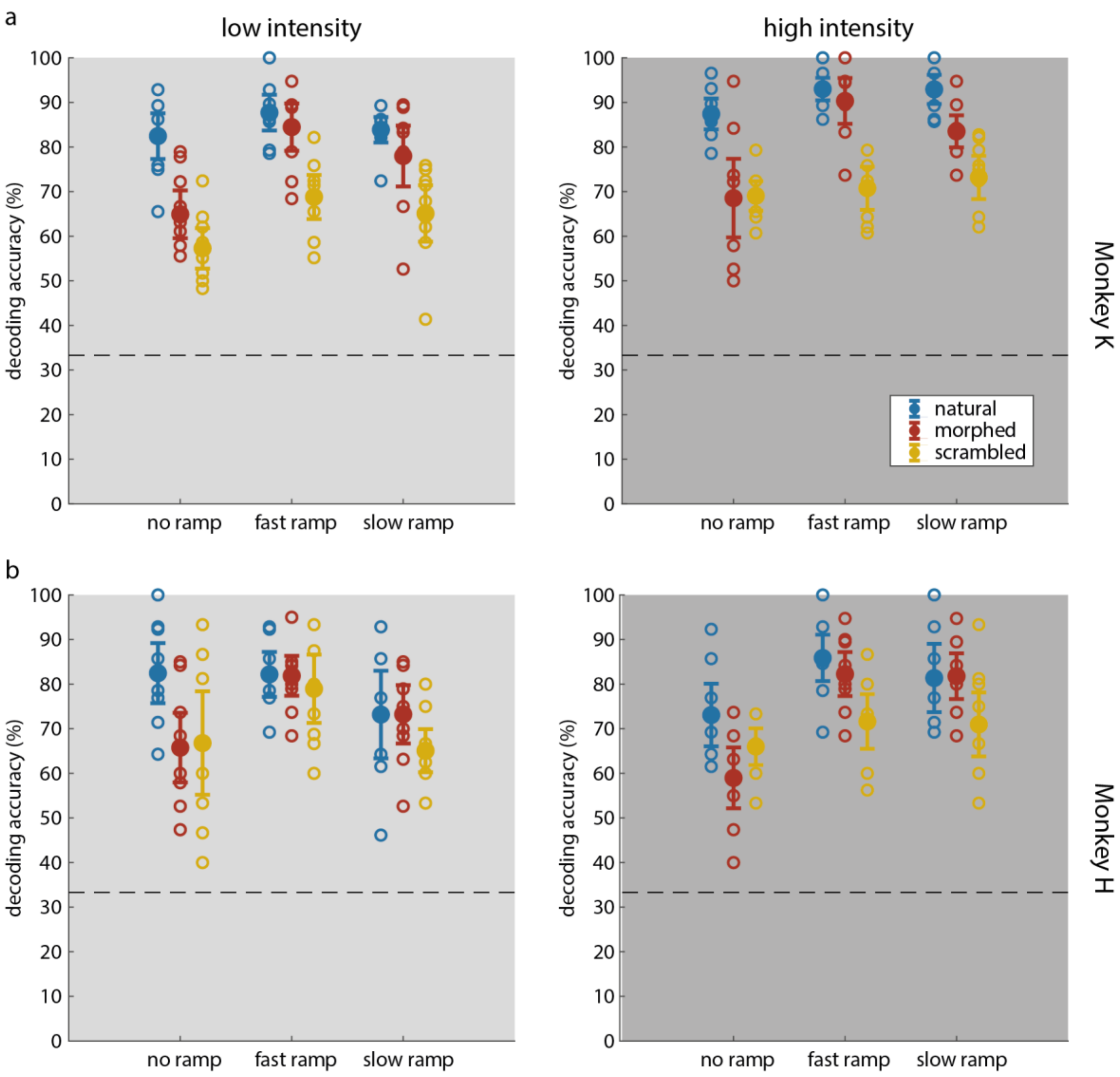

Supplementary Figure 12 Response sequences evoked by natural stimuli are most informative about stimulus identity.

Same conventions as in Figure 3b, but separate for the two monkeys (a. monkey K, b. monkey H). Left and right panels refer to low and high intensity conditions, respectively. Source data are provided as a Source Data file.

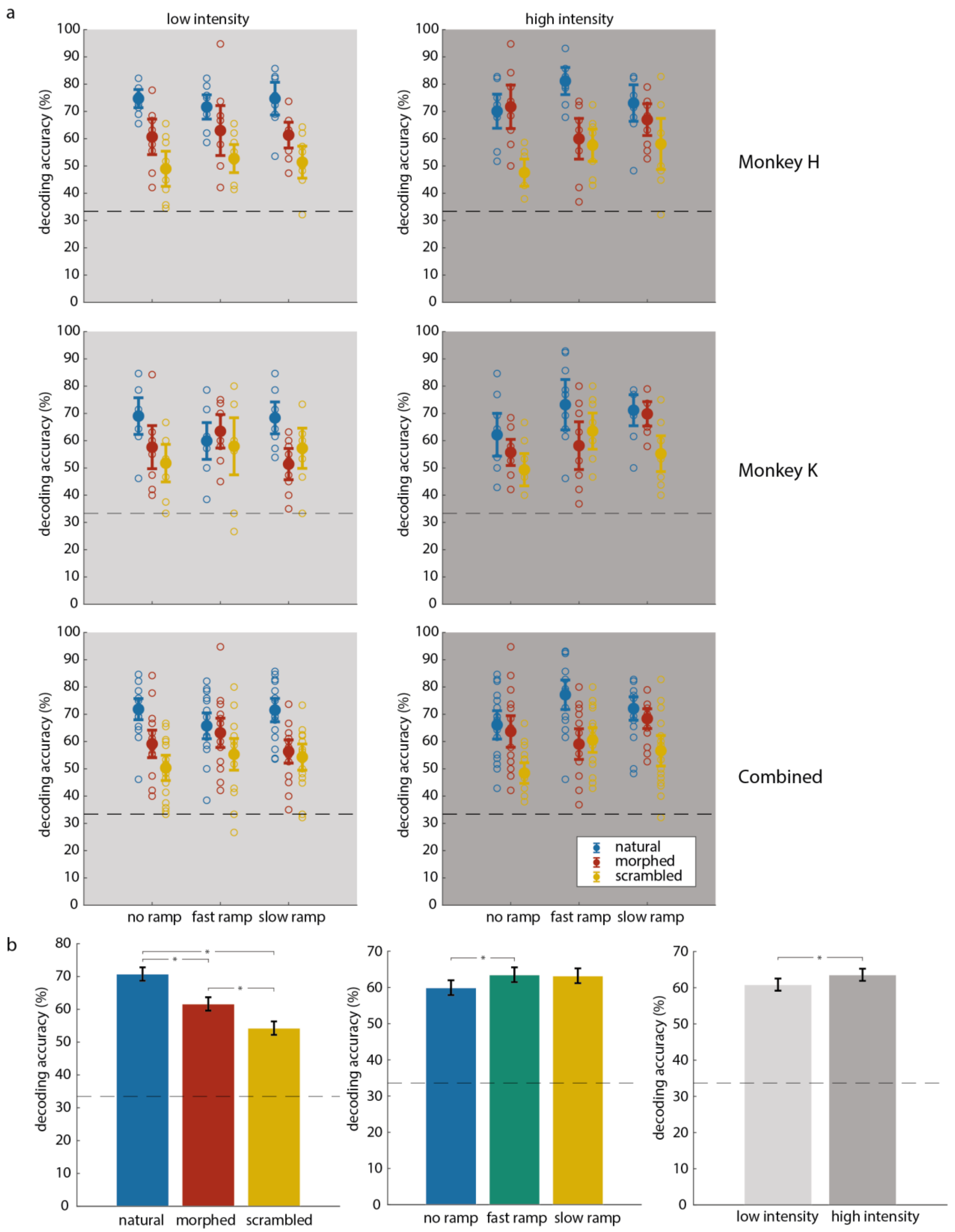

Supplementary Figure 13 Response sequences evoked by natural stimuli are most informative about stimulus identity. Measurements based on response onset latencies instead of the peak response time.

a. Accuracy of decoding stimulus identity from the sequence of response onset latencies. Horizontal dashed lines indicate chance level. Left and right columns denote the two intensity conditions. The first and second rows are the results for

monkey H and K, respectively. The third row shows the pooled results from both monkeys. ANOVA performed on the pooled data showed that there was a significant effect of stimulus structure category ($F_{2,354} = 62.96$, $p < 0.01$), ramp conditions ($F_{2,354} = 3.64$, $p = 0.027$) and maximal plateau intensity ($F_{1, 354} = 5.08$, $p = 0.025$). b. Decoding accuracy marginalized over stimulus structure (left), ramp condition (middle) and maximal plateau intensity (right). For stimulus structure, all pair-wise differences between natural, morphed, and scrambled image conditions were significant ($p < 0.05$, two-sided t-test). For the ramp conditions, the decoding accuracy in the no ramp condition was significantly different from the fast ramp ($p = 0.047$), but not from the slow ramp condition ($p = 0.079$). There was also no difference in accuracy between fast ramp and slow ramp conditions ($p = 1.00$). For the maximal intensity conditions, the decoding accuracy was significantly different between low and high intensity conditions ($p = 0.025$). Bonferroni correction for multiple comparison was applied. All error bars indicate 95% confidence interval around the mean. Source data are provided as a Source Data file.

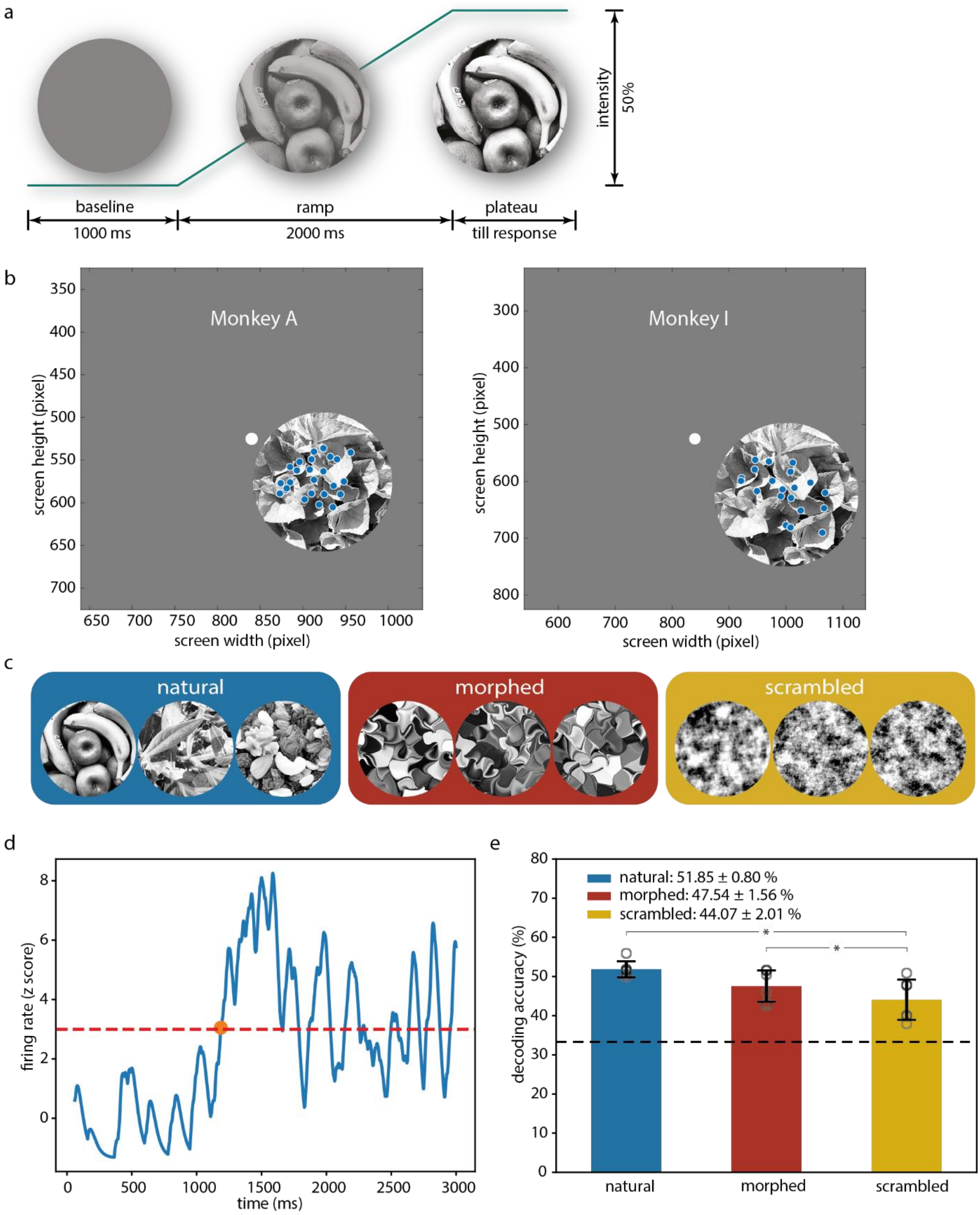

Supplementary Figure 14 Response sequences in V1 contain stimulus-specific information that depends on stimulus category.

a. Paradigm for the V1 experiment. Stimulus intensity (alpha value) increases linearly from 0 to 50% over an interval of 2000 ms. b. Schematic of stimulus position, fixation point (white dot) and V1 classical receptive fields (blue dots) on the display monitor for monkey A (left) and I (right). c. Example stimuli for three structure categories. Original stimulus images cannot be presented due to the journal's license policy. d. Method for determination of response latency. e. Accuracy of decoding

stimulus identity from the sequence of response onset latencies for three stimulus structure categories. There is a significant influence of stimulus category on decoding accuracy (ANOVA, $F = 5.33$, $p = 0.0178$). Asterisks denote statistical significance in post-hoc pairwise comparisons. Horizontal dashed line marks chance level. Error bars indicate 95% confidence interval. Figure legend shows mean ± s.e.m.

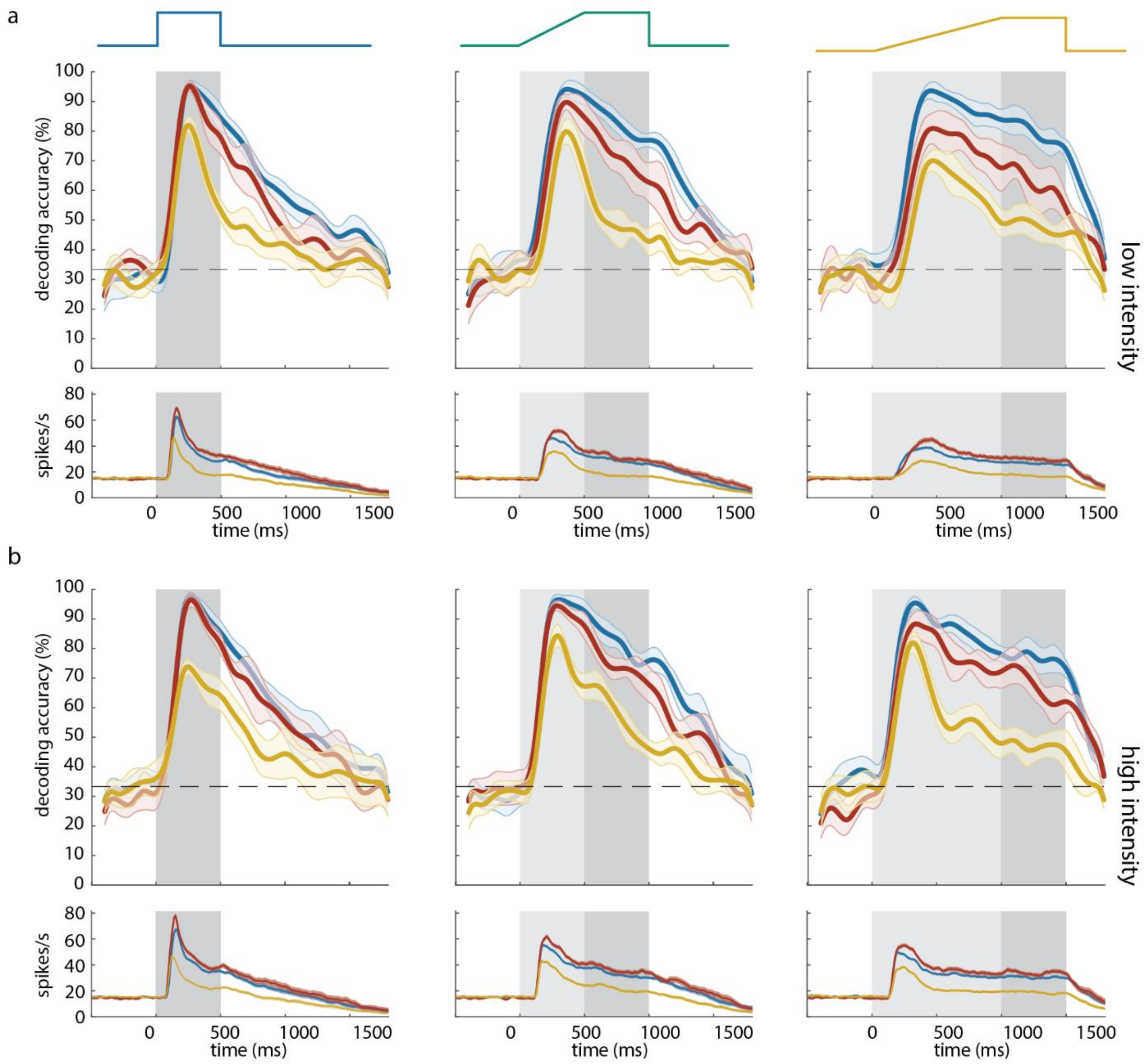

Supplementary Figure 15 Natural stimuli extend the persistence of stimulus-specific information.

Same conventions as in Figure 5 but for monkey K. a. Upper panels: time-resolved decoding accuracy. Lower panels: firing rate. The three columns show the no ramp (left), fast ramp (middle) and slow ramp (right) conditions. b. Same conventions as in a but for the high intensity condition. Source data are provided as a Source Data file.

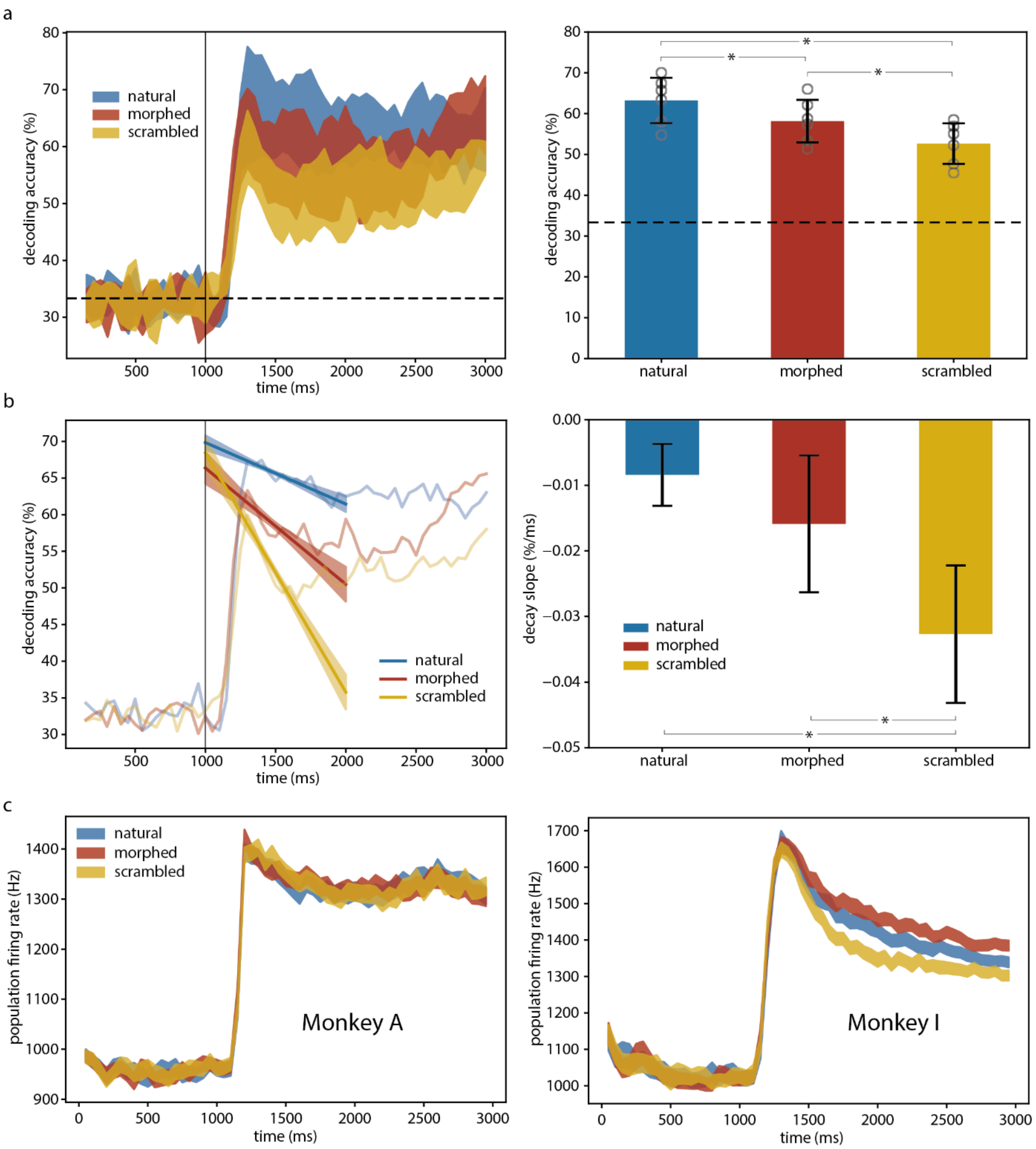

Supplementary Figure 16 Natural stimuli extend the persistence of stimulus-specific information.

Results from area V1.

a. Left: the accuracy of decoding stimulus identity from firing rates over time, for the three stimulus categories. Right: average decoding accuracy over the whole response period for the different stimulus categories (natural: 63.24 ± 2.16 %, morphed: 58.17 ± 2.03 %, scrambled: 52.66 ± 1.93 %; mean ± s.e.m., save below). Asterisks denote $p < 0.05$ (two-sided t-test). b. Left: quantification of decay speed of decoding accuracy. Right: comparison of decay speed for different stimulus categories (natural: $8.4 \pm 2.0 \times 10^{-3}$ %/ms, morphed: $1.59 \pm 0.44\ 10^{-2}$ %/ms, scrambled: $3.27 \pm 0.40\ 10^{-2}$ %/ms). c. Population firing rate (sum across channels) for three stimulus structure categories, for monkey A (left) and I (right). All shaded areas and error bars indicate 95% confidence level around the mean.

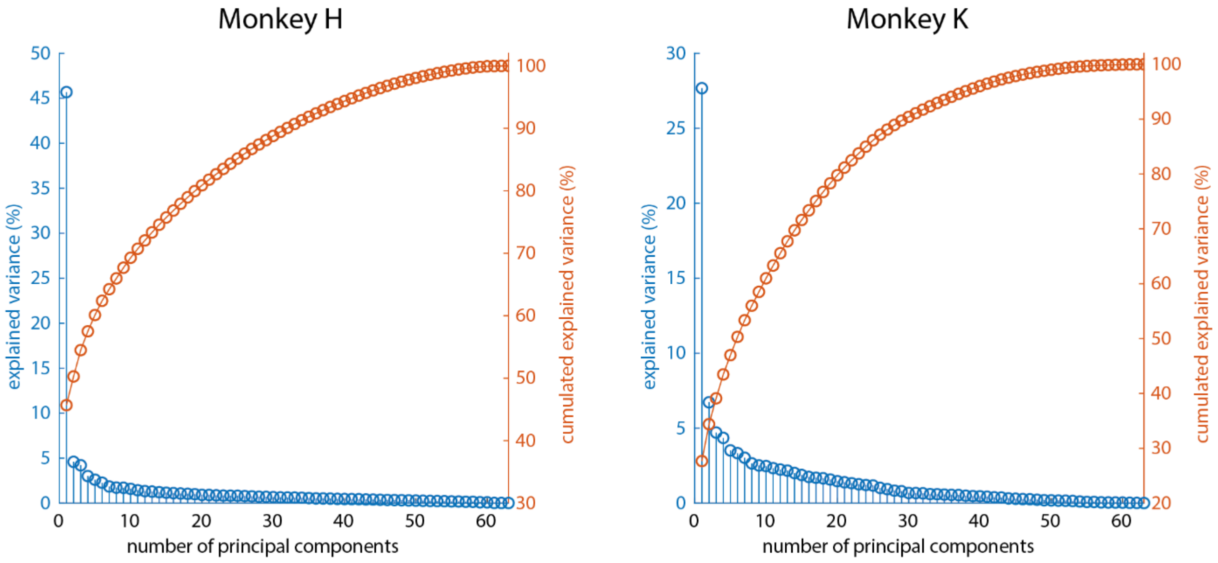

Supplementary Figure 17 Variance explained by principal components. Source data are provided as a Source Data file.

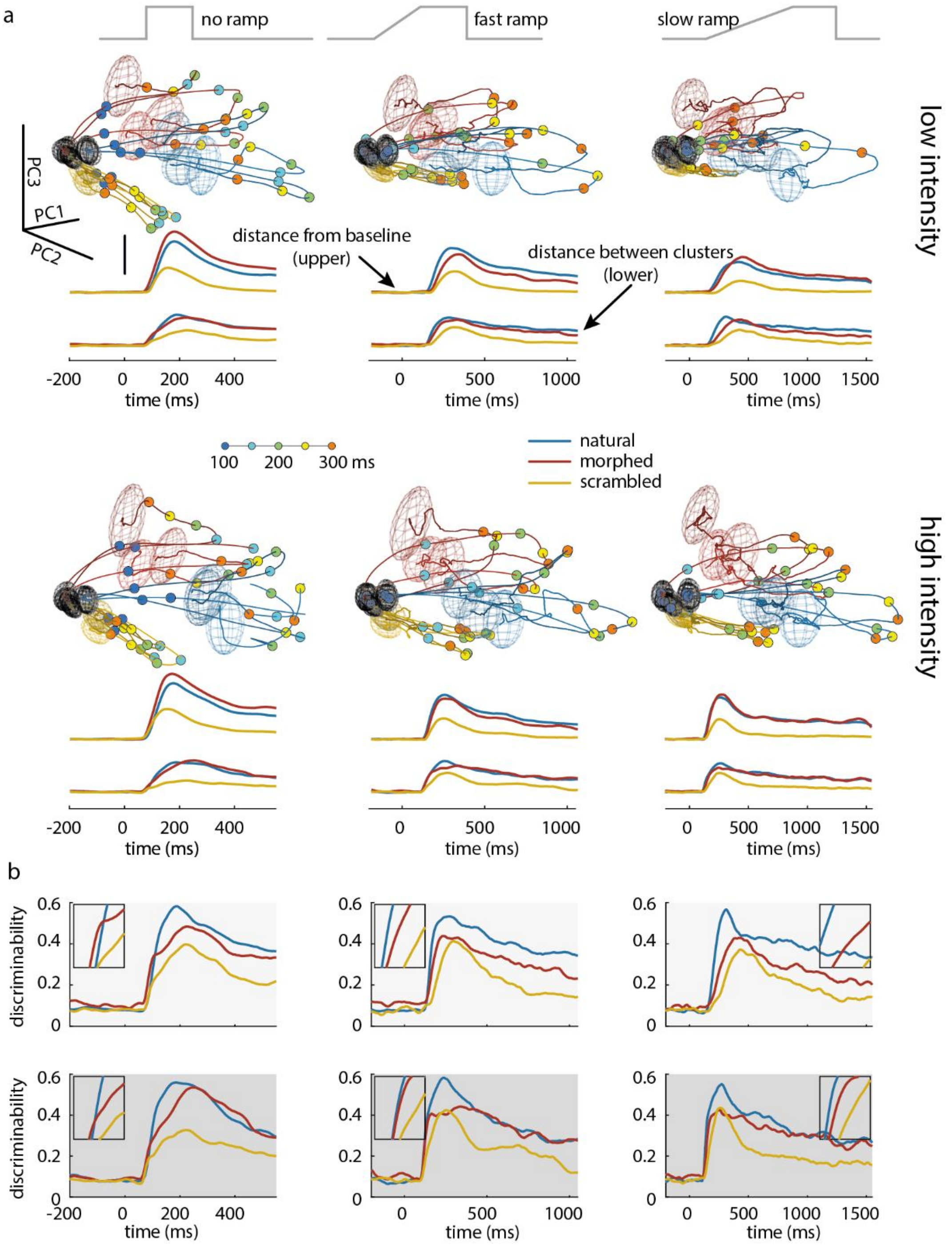

Supplementary Figure 18 Visualization of the time course of stimulus-specific trajectories of neural population activity. a & b: same conventions as in Figure 6, but for monkey K.

Supplementary Notes

Sequential responses imply network interactions.

We performed a number of control analyses to determine whether the sequential responses were due to network interactions or simply reflect differences in the excitability and tuning of the neurons

and their afferent drive (Supplementary Figure 7 and Supplementary Figure 8 for two animals). A trivial cause for stimulus-specific sequences of response latencies could be differences in sensitivity and feature selectivity of the transmission chains feeding the different neurons. As the intensity ramps up, neurons with high sensitivity would respond earlier than those with low sensitivity. In this case, the intensity at which a particular neuron begins to respond should be the same for ramps with different slopes. To evaluate this possibility, we calculated the preferred intensity of each channel, i.e., the intensity at which the firing rate peaked, and then compared the preferred intensity between slow and fast ramp conditions (Supplementary Figure 7a). We found that the preferred intensity was different for the two ramps regardless of intensity (t_{188} = 15.6, p < 0.01 for low intensity, t_{188} = 16.38, p < 0.01 for high intensity), and was lower for the slow ramp condition (Supplementary Figure 7b). Neurons integrate afferent drive over some time interval until their individual firing threshold is reached. Thus, differences between theses integration intervals could also be responsible for the generation of sequences. If this were the case, the integral over the drive should be identical irrespective of the ramp durations. With slow ramps, more time should elapse until firing threshold is reached (Supplementary Figure 7c). We calculated the preferred integrated intensity, i.e., the summed input intensity till the time of peak firing, and compared slow with fast ramp conditions. We found that the preferred integrated intensity was not equal between the two ramps (t_{188} = 3.84, p = 0.00017 for low intensity, t_{188} = 5.56, p < 0.01 for high intensity, Supplementary Figure 7d). This was also the case when ramps were compared that had the same duration but ended at different maximal intensities (Supplementary Figure 7e). Again, the preferred integrated intensity differed for the two intensity conditions (t_{188} = 7.40, p < 0.01 for fast ramp, t_{188} = 6.00, p < 0.01 for slow ramp. Supplementary Figure 7f).

An implicit assumption underlying these controls is that neurons are non-leaky current integrators, which is not the case. However, if we assume in addition the influence of leak currents, the summed input drive would have to be even stronger in the slow rather than the fast ramp condition, and the low rather than the high intensity condition. However, our results point in the opposite direction.

The preferred integrated intensity was systematically lower in the slow ramp (Supplementary Figure 7d) and the low intensity conditions (Supplementary Figure 7f). These results are incompatible with the assumption that the sequences resulted from differences in excitability, tuning or afferent drive. Rather, they suggest that the sequences resulted from network interactions.
